# The telestroke and thrombolysis therapy in diabetic stroke patients

**DOI:** 10.1186/s13098-019-0421-2

**Published:** 2019-05-09

**Authors:** Thomas I. Nathaniel, Chibueze Ubah, Leah Wormack, Jordan Gainey

**Affiliations:** School of Medicine-Greenville, University of South Carolina, Greenville, SC 29605 USA

**Keywords:** Acute stroke, Diabetes mellitus, Exclusion, Inclusion, Telestroke, Non telestroke

## Abstract

**Objective:**

Several controversial findings have been reported on treatment outcomes for diabetic stroke patients that received thrombolysis therapy in the hospital. We determined whether the association between telestroke technology, thrombolysis therapy and clinical risk factors in diabetic acute ischemic stroke may result in the inclusion or exclusion or more diabetic ischemic stroke patients for thrombolysis therapy.

**Methods:**

Retrospective data that comprises of a total of 3202 acute ischemic stroke patients from a regional stroke registry that contained telestroke and non telestroke patients with a primary diagnosis of acute ischemic stroke of which 312 were identified as diabetic stroke patients were used in this study. Multivariate logistic regression models were used to determine the associated pre-clinical risk factors, and demographics associated with recombinant tissue plasminogen activator (rtPA) therapy in a subset of diabetic acute ischemic stroke patients in the telestroke and non-telestroke settings.

**Results:**

In the telestroke, only higher International Normalized Ratio (INR) [odds ratio, OR = 0.063 (0.003–1.347, 95% confidence interval (CI)] was associated with exclusion from thrombolysis. Direct admission [OR, 3.141 (1–9.867), 95% CI] and telestroke [OR, 4.87 (1.834–12.928), 95% CI] were independent predictors in the inclusion for thrombolysis therapy. In the non telestroke, older age (> 80 years) [(OR), 0.955 (0.922–0.989), 95% CI], higher blood glucose level [OR, 0.994 (0.99–0.999); 95% CI], higher INR [OR, 0.113 (0.014–0.944); 95% CI], and renal insufficiency [OR, 0.163 (0.033–0.791); 95% CI] were associated with exclusion while higher NIH stroke scale [OR, 1.068 (1.009–1.13); 95% CI] was associated with inclusion for thrombolysis in the non telestroke.

**Conclusion:**

The non-telestroke setting admitted more diabetic stroke patients to the hospital, but more were excluded from thrombolysis therapy when compared with the telestroke setting. Measures to improve clinical risk factors that excluded more diabetic ischemic stroke patients in the non telestroke will improve the use of thrombolysis in the treatment of diabetic acute ischemic stroke patients.

## Introduction

Diabetes mellitus is a frequently identified comorbid risk factor in acute ischemic stroke. The risk of ischemic stroke in diabetic patients is twofold higher when compared to people without diabetes [1]. This underlies the close relationship between these two co-occurring common diseases. Though the disease processes are closely related, controversial findings have been reported on treatment outcomes for diabetic stroke patients that received thrombolysis therapy [2–6]. This is because the management of diabetic stroke patients is complicated, and this results in most of the observed controversial outcomes.

Although diabetes is not an absolute or relative exclusion criteria for thrombolysis, a low rate of thrombolysis therapy has been reported in diabetic ischemic stroke patients due to concerns over poorer outcomes [7]. Proposed factors for the poor response include stroke severity [8], a higher risk of developing post stroke hyperglycemia [9] and vascular risk factors [2]. Thrombolysis is known to produce better outcomes in stroke patients when compared with diabetic stroke patients [6], and clinical trials [10] did not suggest the withholding of thrombolysis therapy from diabetic stroke patients [11]. Moreover, existing studies suggest that the lower rate of thrombolysis therapy in diabetic stroke patients does not appear to be related to contraindications for thrombolysis because a comparison of contraindications for thrombolysis between ischemic stroke patients with and without diabetes did not reveal a significant difference [12].

It has been shown that a practice-based model of telestroke can manage pretreatment clinical risk factors for thrombolysis therapy relaxing the criteria for the inclusion or exclusion for thrombolysis in ischemic stroke patients [13]. Although the telestroke is known with favorable outcomes in acute ischemic stroke [13–17], however, the effect of telestroke technology in enhancing the use of thrombolysis therapy in diabetic stroke patients when compared with treatment is not known. We know that several studies in non telestroke settings, reveal controversial findings on treatment outcomes for diabetic stroke patients that received thrombolysis therapy. While some studies report poorer outcomes in diabetic ischemic stroke patients when compared with non-diabetic acute ischemic stroke patients [2–4], others have shown the safety and beneficial effects of recombinant tissue plasminogen activator (rtPA) [5, 6]. It is also known that treatment outcomes in telestroke programs have been favorable, and consistent with good expectations in several studies in acute ischemic stroke [13–19]. What is not known is whether the association between telestroke technology, thrombolysis therapy and clinical risk factors in diabetic acute ischemic stroke may result in the inclusion or exclusion of diabetic ischemic stroke patients for thrombolysis therapy. We investigated this issue in a population of diabetic acute ischemic stroke patients treated in a telestroke and compared our findings with a non telestroke setting. We used multivariate models to predict the odds of including more diabetic stroke patients for thrombolysis therapy in the telestroke when compared with the non telestroke setting. The current study investigated telestroke technology in the use of thrombolysis therapy in diabetic acute ischemic stroke patients with various baseline clinical risk factors.

## Method

### Patient selection and baseline characteristics

Retrospective data were collected from the acute ischemic stroke registry of Greenville Health System (GHS) between January 2010 and June 2016. The registry has been described in our previous studies [20–23]. Patients were selected with prospective inclusion of consecutive patients with diabetic acute ischemic stroke treated in a stroke center (non-telestroke) and telestroke network. Data for the various pre-clinical risk factors was extracted including; atrial fib/flutter, carotid artery stenosis, congestive heart failure, depression, dyslipidemia, coronary artery disease, family history of stroke, hormone replacement therapy, hypertension, migraine, obesity, peripheral vascular disease, previous stroke, previous TIA, prosthetic heart valve, renal insufficiency, sleep apnea, smoking, substance abuse. Additional variables from time of admission were also included. The National Institutes of Health stroke scale (NIHSS) was used to evaluate severity of neurologic impairment. Laboratory analysis for the concentrations of total cholesterol, low-density lipoprotein cholesterol (LDL), triglycerides, lipids, high-density lipoprotein cholesterol (HDL), blood glucose and creatine were obtained at admission. Values for systolic blood pressure, diastolic blood pressure and International Normalized Ratio (INR) were determined.

Upon admission, all patients underwent brain computed tomography. Patients with subarachnoid and intracerebral hemorrhage were excluded in our analysis. A standardized stroke protocol was used in all patients, including T2-weighted, T1-weighted, and diffusion-weighted images. Data on symptom onset time and the admission to Emergency Department (ED) for both telestroke and non telestroke diabetic stroke patients were collected. Patients that were directly admitted to the ED or with emergency medical services (EMS) and those with indirect admission by being transferred to the ED in the telestroke or non telestroke from another hospital were also identified. Data on patient demographics, including age, sex, race, and ethnicity were also extracted Information on the ambulation status prior to event, during and at discharge were also collected. Ethical approval was obtained from the Institutional Review Board of Greenville Health System and the institutional Committee for Ethics.

### Data analysis

The SPSS package version 20 (Chicago, IL, USA) for Windows was used for all statistical analyses and P < 0.05 was used to establish statistical significance for all comparisons between groups. We used univariate analysis to analyze baseline characteristics including age, gender, medical history, prestroke treatments and admission parameters such as blood glucose and stroke severity. This allowed us to determine baseline or pre-clinical risk factors that were associated with inclusion or exclusion for recombinant tissue plasminogen activator (rtPA). All discrete variables were represented as number (percentage) and comparisons between groups were made using Pearson’s Chi Squared analyses. Descriptive statistics were calculated for the demographic and clinical characteristics of patients. All continuous variables were presented as mean (STD), and comparisons between groups were determined using the Student’s *T* test. All variables presented in Tables 1 and 2 were analyzed using univariate analysis while multivariate models were used to identify significant associations with exclusion or inclusion for thrombolysis therapy in the whole diabetic stroke population in telestroke and non telestroke settings (see Tables 3, 4 and 5). Adjustments in the multivariate analysis were based on univariate significance. Subsequent multivariate logistic regression was based on risk factors in diabetic stroke patients associated with thrombolysis therapy and specific for telestroke or non telestroke identified by the univariate analysis. This analysis identified independent predictors of exclusion or inclusion for thrombolysis therapy. The multivariable model was built by stepwise conditional logistic regression. We used a backward procedure as a follow-up to test the modeling strategy, while the test for the log likelihood was used to assess the suitability of fit and to compare nested models. All variables that produced changes > 10% of the odds ratio (OR) when eliminated were considered to be confounding variables, while variables with a value of P < 0.01 on univariate testing were included. All stepwise regression models were assessed using Hosmer & Lemeshow test, Cox & Snell R^2^ and Classification Plots. Multicollinearity of variables were assessed with variance inflation factor analysis to confirm independence of variables included in regression model.

**Table 1:** Demographic factors and clinical characteristics of acute ischemic stroke patients with a history of diabetes divided by telestroke status

Characteristic	Non-telestroke	Telestroke	P-value
	(N = 180)	(N = 132)	
Patient age in years			
Mean ± SD	69.3 ± 12.7	65.9 ± 12.3	0.020*
Age group: no. (%)			
< 50 years	14 (7.8)	11 (8.3)	0.069
50–59	26 (14.4)	20 (15.2)	
60–69	43 (23.9)	49 (37.1)	
70–79	54 (30)	33 (25)	
≥ 80	43 (23.9)	19 (14.4)	
Gender: no. (%)			
Male	88 (48.9)	69 (52.3)	0.555
Female	92 (51.1)	63 (47.7)	
Race: no. (%)			
Caucasian	127 (70.6)	102 (77.3)	0.212
African-American	32 (17.8)	19 (14.4)	
Other	3 (1.7)	3 (2.3)	
Hispanic ethnicity: no. (%)	5 (2.8)	6 (4.5)	0.403
Body mass index			
Mean ± SD	29.5 ± 7.3	32.2 ± 7.5	0.001*
Medical history: no. (%)			
Atrial fib/flutter	39 (21.7)	11 (8.3)	0.002*
Carotid artery stenosis	12 (6.7)	6 (4.5)	0.427
Congestive heart failure	28 (15.6)	17 (12.9)	0.506
Coronary artery disease	80 (44.4)	62 (47)	0.658
Depression	1 (0.6)	27 (20.5)	< 0.001*
Dyslipidemia	124 (68.9)	92 (69.7)	0.879
Family history of stroke	14 (7.8)	24 (18.2)	0.006*
Hormone replacement therapy	3 (1.7)	3 (2.3)	0.7
Hypertension	165 (91.7)	123 (93.2)	0.62
Migraine	5 (2.8)	4 (3)	0.895
Obesity	78 (43.3)	85 (64.4)	< 0.001*
Peripheral vascular disease	23 (12.8)	11 (8.3)	0.213
Previous stroke	73 (40.6)	31 (23.5)	0.002*
Previous TIA	22 (12.2)	9 (6.8)	0.115
Prosthetic heart valve	1 (0.6)	0 (0)	0.391
Renal insufficiency	24 (13.3)	9 (6.8)	0.065
Sleep apnea	0 (0)	11 (8.3)	< 0.001*
Smoking	41 (22.8)	25 (18.9)	0.412
Substance abuse	5 (2.8)	2 (1.5)	0.457
Initial NIH stroke scale			
Mean ± SD	10.8 ± 8.6	8.9 ± 7.6	0.063
Initial labs and vitals			
Total cholesterol	165.7 ± 56.9	165.1 ± 43.4	0.885
Triglycerides	157.3 ± 118.4	159.3 ± 106.1	0.307
HDL	39.4 ± 12.9	37.8 ± 11.7	0.565
LDL	95.8 ± 36.5	98.4 ± 35.3	0.889
Lipids	7.6 ± 2.1	7.6 ± 2.1	0.067
Blood glucose	195.8 ± 115.2	173 ± 97	0.054
Creatinine	1.5 ± 1.1	1.2 ± 1	0.007*
INR	1.1 ± 0.3	1 ± 0.2	0.074
Heart rate	84.1 ± 19.2	80.4 ± 16.9	0.097
Systolic blood pressure	155.9 ± 33.2	150.6 ± 23.6	0.158
Diastolic blood pressure	81.8 ± 19.2	78.8 ± 17.1	<0.001*
Medications prior to admission: no. (%)			
Antiplatelet or anticoagulant	112 (62.2)	79 (59.8)	0.671
Antihypertensive	150 (83.3)	114 (86.4)	0.464
Cholesterol reducer	113 (62.8)	91 (68.9)	0.258
Diabetic medication	128 (71.1)	100 (75.8)	0.361
Ambulation status prior to event: no. (%)			
Ambulate independently	148 (82.2)	121 (91.7)	0.106
Ambulate with assistance	12 (6.7)	3 (2.3)	
Unable to ambulate	11 (6.1)	5 (3.8)	
Not documented	9 (5)	3 (2.3)	
Ambulation status on admission: no. (%)			
Ambulate independently	20 (11.1)	26 (19.7)	0.016*
Ambulate with assistance	18 (10)	23 (17.4)	
Unable to ambulate	75 (41.7)	39 (29.5)	
Not documented	67 (37.2)	44 (33.3)	
Ambulation status on discharge: no. (%)			
Ambulate independently	69 (38.3)	61 (46.2)	0.044*
Ambulate with assistance	46 (25.6)	42 (31.8)	
Unable to ambulate	47 (26.1)	18 (13.6)	
Not documented	18 (10)	11 (8.3)	
First care received: no. (%)			
Emergency department	159 (88.3)	38 (28.8)	< 0.001*
Direct admission	21 (11.7)	94 (71.2)	
rtPA administration	68 (37.8)	114 (86.4)	< 0.001*
Improved ambulation	109 (60.6)	89 (67.4)	0.213

Continuous variables are represented as Mean ± S.D. and comparisons between groups are made with a Student’s T Test. Discrete variables are represented as Count (Percent Frequency) and comparisons between groups were made using Pearson’s Chi Squared

*P < 0.05

**Table 2:** Clinical characteristics, medical history, and presenting symptoms of acute ischemic stroke patients with a history of diabetes stratified by rtPA status and telestroke status

Characteristic	Non-telestroke			Telestroke		
	No rtPA	rtPA	P-value	No rtPA	rtPA	P-value
	(N = 112)	(N = 68)		(N = 18)	(N = 114)	
Patient age in years						
Mean ± SD	70.8 ± 12	66.8 ± 13.5	0.043*	66.9 ± 13.2	65.8 ± 12.2	0.727
Age group: no. (%)						
< 50 years	6 (5.4)	8 (11.8)	0.014	1 (5.6)	10 (8.8)	0.362
50–59	16 (14.3)	10 (14.7)		5 (27.8)	15 (13.2)	
60–69	20 (17.9)	23 (33.8)		5 (27.8)	44 (38.6)	
70–79	42 (37.5)	12 (17.6)		3 (16.7)	30 (26.3)	
≥ 80	28 (25)	15 (22.1)		4 (22.2)	15 (13.2)	
Gender: no. (%)						
Male	49 (43.8)	39 (57.4)	0.077	9 (50)	60 (52.6)	0.835
Female	63 (56.3)	29 (42.6)		9 (50)	54 (47.4)	
Race: no. (%)				(0)	(0)	
Caucasian	73 (65.2)	54 (79.4)	0.6	14 (77.8)	88 (77.2)	0.74
African-American	21 (18.8)	11 (16.2)		2 (11.1)	17 (14.9)	
Other	3 (2.7)	0 (0)		0 (0)	3 (2.6)	
Hispanic ethnicity: no. (%)	2 (1.8)	3 (4.4)	0.917	0 (0)	6 (5.3)	0.319
Body mass index						
Mean ± SD	29.6 ± 7.6	29.1 ± 7	0.657	31 ± 7	32.4 ± 7.6	0.447
Medical history: no. (%)						
Atrial fib/flutter	30 (26.8)	9 (13.2)	0.032	2 (11.1)	9 (7.9)	0.646
Carotid artery stenosis	10 (8.9)	2 (2.9)	0.118	0 (0)	6 (5.3)	0.319
Congestive heart failure	21 (18.8)	7 (10.3)	0.129	4 (22.2)	13 (11.4)	0.203
Coronary artery disease	48 (42.9)	32 (47.1)	0.582	9 (50)	53 (46.5)	0.782
Depression	1 (0.9)	0 (0)	0.435	3 (16.7)	24 (21.1)	0.668
Dyslipidemia	76 (67.9)	48 (70.6)	0.701	13 (72.2)	79 (69.3)	0.802
Family history of stroke	8 (7.1)	6 (8.8)	0.683	0 (0)	24 (21.1)	0.031
Hormone replacement therapy	2 (1.8)	1 (1.5)	0.873	0 (0)	3 (2.6)	0.486
Hypertension	103 (92)	62 (91.2)	0.853	16 (88.9)	107 (93.9)	0.437
Migraine	1 (0.9)	4 (5.9)	0.048	1 (5.6)	3 (2.6)	0.501
Obesity	48 (42.9)	30 (44.1)	0.869	12 (66.7)	73 (64)	0.828
Peripheral vascular disease	17 (15.2)	6 (8.8)	0.216	1 (5.6)	10 (8.8)	0.646
Previous stroke	50 (44.6)	23 (33.8)	0.152	5 (27.8)	26 (22.8)	0.644
Previous TIA	13 (11.6)	9 (13.2)	0.746	2 (11.1)	7 (6.1)	0.437
Prosthetic heart valve	1 (0.9)	0 (0)	0.435	(0)	(0)	
Renal insufficiency	20 (17.9)	4 (5.9)	0.022	1 (5.6)	8 (7)	0.819
Smoking	23 (20.5)	18 (26.5)	0.357	4 (22.2)	21 (18.4)	0.702
Substance abuse	2 (1.8)	3 (4.4)	0.299	0 (0)	2 (1.8)	0.571
Initial NIH stroke scale						
Mean ± SD	10.3 ± 9.2	11.3 ± 7.8	0.462	10.7 ± 9.4	8.6 ± 7.4	0.34
Initial labs and vitals						
Total cholesterol	168.1 ± 62.8	162.4 ± 48.4	0.547	160.9 ± 53	165.7 ± 42	0.682
Triglycerides	157.6 ± 125.1	157 ± 109.7	0.976	128.4 ± 68.7	163.9 ± 110.1	0.213
HDL	40.2 ± 14.1	38.3 ± 11	0.379	38.4 ± 13.5	37.8 ± 11.5	0.843
LDL	95.2 ± 34.3	96.6 ± 39.4	0.816	98.9 ± 50.6	98.3 ± 32.7	0.945
Lipids	7.8 ± 2.2	7.3 ± 1.9	0.171	7.6 ± 2.4	7.6 ± 2.1	0.945
Blood glucose	210.9 ± 126.8	171 ± 88.3	0.014*	184.2 ± 133.1	171.3 ± 90.6	0.602
Creatinine	1.6 ± 1.3	1.3 ± 0.7	0.032*	1.6 ± 2.3	1.2 ± 0.6	0.375
INR	1.2 ± 0.4	1.1 ± 0.1	0.002*	1.2 ± 0.5	1 ± 0.1	0.347
Heart rate	85.5 ± 21.2	81.8 ± 15.2	0.216	77.9 ± 15.4	80.8 ± 17.1	0.505
Systolic blood pressure	155.1 ± 35	157.3 ± 30.1	0.669	151.7 ± 22	150.4 ± 24	0.835
Diastolic blood pressure	82.5 ± 20.6	80.7 ± 16.8	0.555	77.8 ± 14.4	79 ± 17.5	0.779
Medications prior to admission: no. (%)						
Antiplatelet or anticoagulant	72 (64.3)	40 (58.8)	0.464	12 (66.7)	67 (58.8)	0.525
Antihypertensive	93 (83)	57 (83.8)	0.891	14 (77.8)	100 (87.7)	0.253
Cholesterol reducer	70 (62.5)	43 (63.2)	0.921	15 (83.3)	76 (66.7)	0.156
Diabetic medication	78 (69.6)	50 (73.5)	0.577	12 (66.7)	88 (77.2)	0.333
Ambulation status prior to event: no. (%)				(0)	(0)	
Ambulate independently	85 (75.9)	63 (92.6)	0.028*	15 (83.3)	106 (93)	0.511
Ambulate with assistance	11 (9.8)	1 (1.5)		1 (5.6)	2 (1.8)	
Unable to ambulate	8 (7.1)	3 (4.4)		(0)	4 (3.5)	
Not documented	8 (7.1)	1 (1.5)		(0)	2 (1.8)	
Ambulation status on admission: no. (%)				(0)	(0)	
Ambulate independently	16 (14.3)	4 (5.9)	0.107	6 (33.3)	20 (17.5)	0.016*
Ambulate with assistance	14 (12.5)	4 (5.9)		0 (0)	23 (20.2)	
Unable to ambulate	45 (40.2)	30 (44.1)		9 (50)	30 (26.3)	
Not documented	37 (33)	30 (44.1)		3 (16.7)	41 (36)	
Ambulation status on discharge: no. (%)						
Ambulate independently	39 (34.8)	30 (44.1)	0.223	9 (50)	52 (45.6)	0.328
Ambulate With assistance	29 (25.9)	17 (25)		3 (16.7)	39 (34.2)	
Unable to ambulate	29 (25.9)	18 (26.5)		3 (16.7)	15 (13.2)	
Not documented	15 (13.4)	3 (4.4)		3 (16.7)	8 (7)	
First care received: no. (%)						
Emergency department	100 (89.3)	59 (86.8)	0.609	12 (66.7)	26 (22.8)	< 0.001*
Direct admission	12 (10.7)	9 (13.2)		6 (33.3)	88 (77.2)	
Improved ambulation	63 (56.3)	46 (67.6)	0.129	14 (77.8)	75 (65.8)	0.313

Continuous variables are represented as Mean ± S.D. and comparisons between groups are made with a Student’s T Test. Discrete variables are represented as Count (Percent Frequency) and comparisons between groups were made using Pearson’s Chi Squared

*P < 0.05

**Table 3:** A stepwise regression model to elucidate clinical factors more associated rtPA inclusion in the total study population of diabetic acute ischemic stroke patients

	B value	Adj. odds ratio	Wald	P value
INR	− 1.971	0.139 (0.029–0.67)	6.054	0.014*
Congestive heart failure	− 1.111	0.329 (0.124–0.878)	4.930	0.026*
Direct admission	1.145	3.141 (1–9.867)	3.842	0.050
Telestroke	1.583	4.87 (1.834–12.928)	10.097	0.001*
Constant	2.256	9.541	6.418	0.011*

Positive B values (Adj, OR > 1) denote variables more associated with rtPA inclusion while negative B values (Adj. OR < 1) denote variables more associated with rtPA exclusion. Multicollinearity and interactions among independent variables were checked. Hosmer–Lemeshow test (P = 0.084), Cox & Snell (R^2^ = 0.260), classification table (overall correctly classified percentage = 74.3%) were applied to check the model fitness

*P < 0.05

**Table 4:** A stepwise regression model to elucidate clinical factors more associated rtPA inclusion in the non-telestroke population

	B value	Adj. odds ratio	Wald	P value
Higher age	− 0.046	0.955 (0.922–0.989)	6.797	0.009*
NIH stroke scale	0.066	1.068 (1.009–1.13)	5.190	0.023*
Blood glucose level	− 0.006	0.994 (0.99–0.999)	6.037	0.014*
INR	− 2.180	0.113 (0.014–0.944)	4.054	0.044*
Renal insufficiency	− 1.817	0.163 (0.033–0.791)	5.064	0.024*
Constant	6.225	505.460	11.330	0.001*

Positive B values (Adj, OR > 1) denote variables more associated with rtPA inclusion while negative B values (Adj. OR < 1) denote variables more associated with rtPA exclusion. Multicollinearity and interactions among independent variables were checked. Hosmer–Lemeshow test (P = 0.493), Cox & Snell (R^2^ = 0.224), classification table (overall correctly classified percentage = 70.8%) were applied to check the model fitness

*P < 0.05

**Table 5:** A stepwise regression model to elucidate clinical factors more associated rtPA inclusion in the telestroke population

	B value	Adj. odds ratio	Wald	P value
INR	− 2.758	0.063 (0.003–1.347)	3.130	0.077
Constant	5.155	173.322	8.724	0.003*

Positive B values (Adj, OR > 1) denote variables more associated with rtPA inclusion while negative B values (Adj. OR < 1) denote variables more associated with rtPA exclusion. Multicollinearity and interactions among independent variables were checked. Cox & Snell (R^2^ = 0.051), and a classification table (overall correctly classified percentage = 91.0%) were applied to check the model fitness

*P < 0.05

## Results

A total of 3202 acute ischemic stroke patients were collected from the stroke registry, 312 were identified as diabetic stroke patients. Of the 312, 180 were in the non-telestroke setting and 132 in the telestroke setting. Comparisons between the baseline demographic and clinical characteristics of telestroke and non-telestroke diabetic acute ischemic stroke patients are presented in Table 1. Telestroke patients tended to be younger (65.9 ± 12.3 vs. 69.3 ± 12.7), have a higher body mass index (32.2 ± 7.5 vs. 29.5 ± 7.3), less likely to have a history of atrial fibrillation (8.3% vs. 21.7%), or a previous stroke (23.5% vs. 40.6%), more likely to have a family history of stroke (18.2% vs. 7.8%) and obese (64.4% vs. 43.3%). At the time of presentation, telestroke patients had a lower creatinine (1.2 ± 1.0 vs. 1.5 ± 1.1) and lower diastolic blood pressure (78.8 ± 17.1 vs. 81.8 ± 19.2). Telestroke patients tended to have a better ambulatory status at baseline, at the time of presentation and at discharge. Telestroke patients were more likely to be directly admitted (71.2% vs. 11.7%) and more likely to receive rtPA (86.4% vs. 37.8%). Multivariate analysis reveals three factors more associated with telestroke patients than non-telestroke patients: obesity [OR, 2.493 (1.135–5.475); 95% CI, P = 0.023], direct admission [OR, 14.248 (6.012–33.766); 95% CI, P < 0.001], and rtPA administration obesity [OR, 1.068 (1.009–1.13); 95% CI, P < 0.001].

**Fig. 1:**
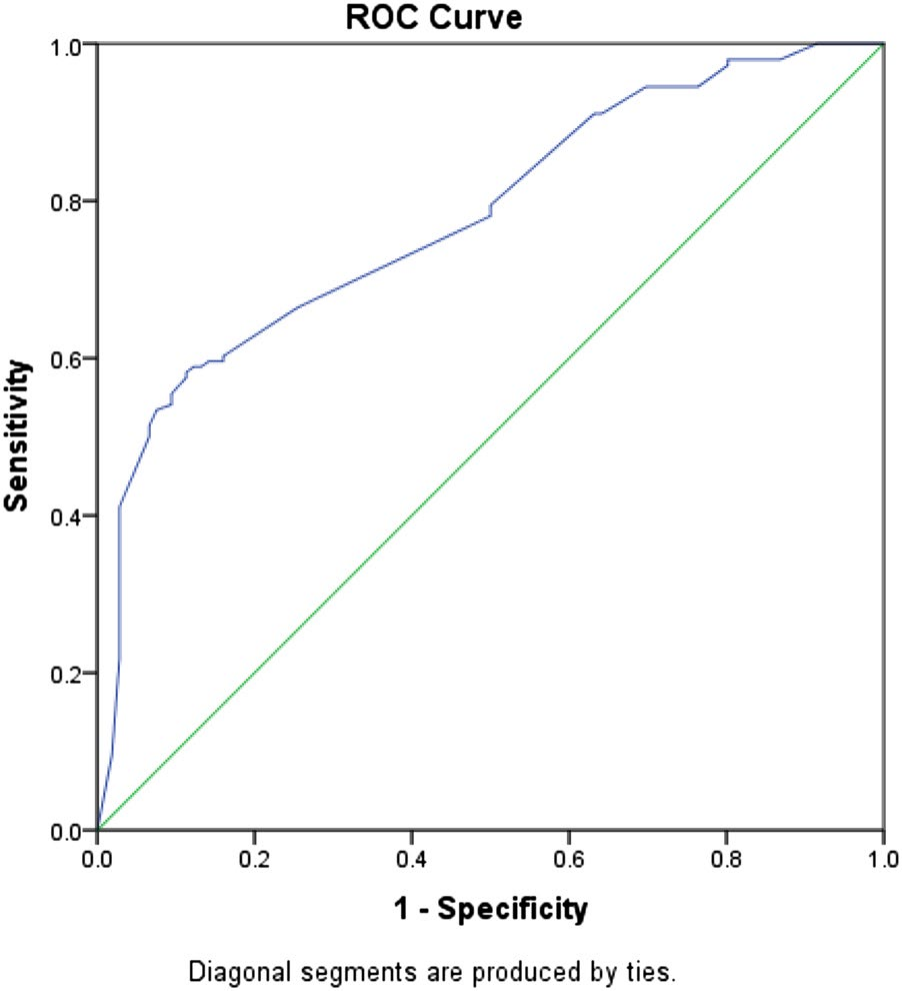
ROC curve to analyze the predictive power of the logistic regression presented in Table 3. The fig indicates AUROC = 0.774 (0.712–0.836) for clinical factors associated rtPA inclusion or exclusion in the non-telestroke population

**Fig. 2:**
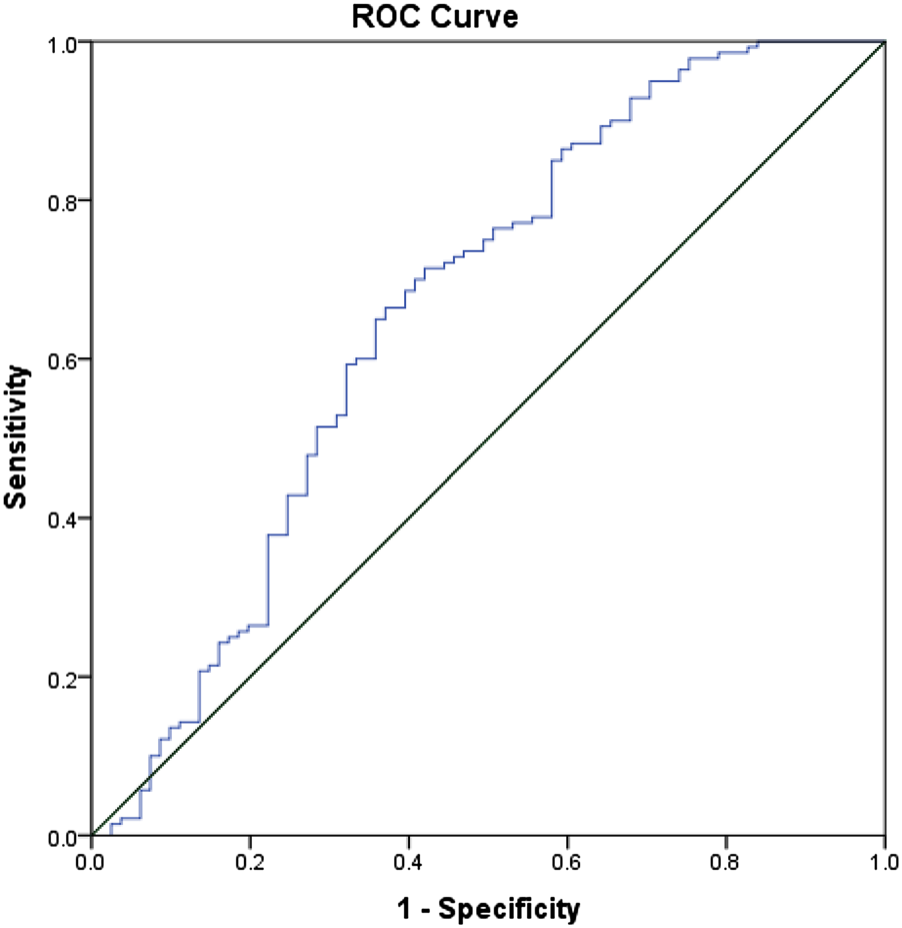
ROC curve to analyze the predictive power of the logistic regression presented in Table 4. The fig indicates AUROC = 0.661 (0.582–0.741) for clinical factors associated rtPA inclusion or exclusion in the non-telestroke population

**Fig. 3:**
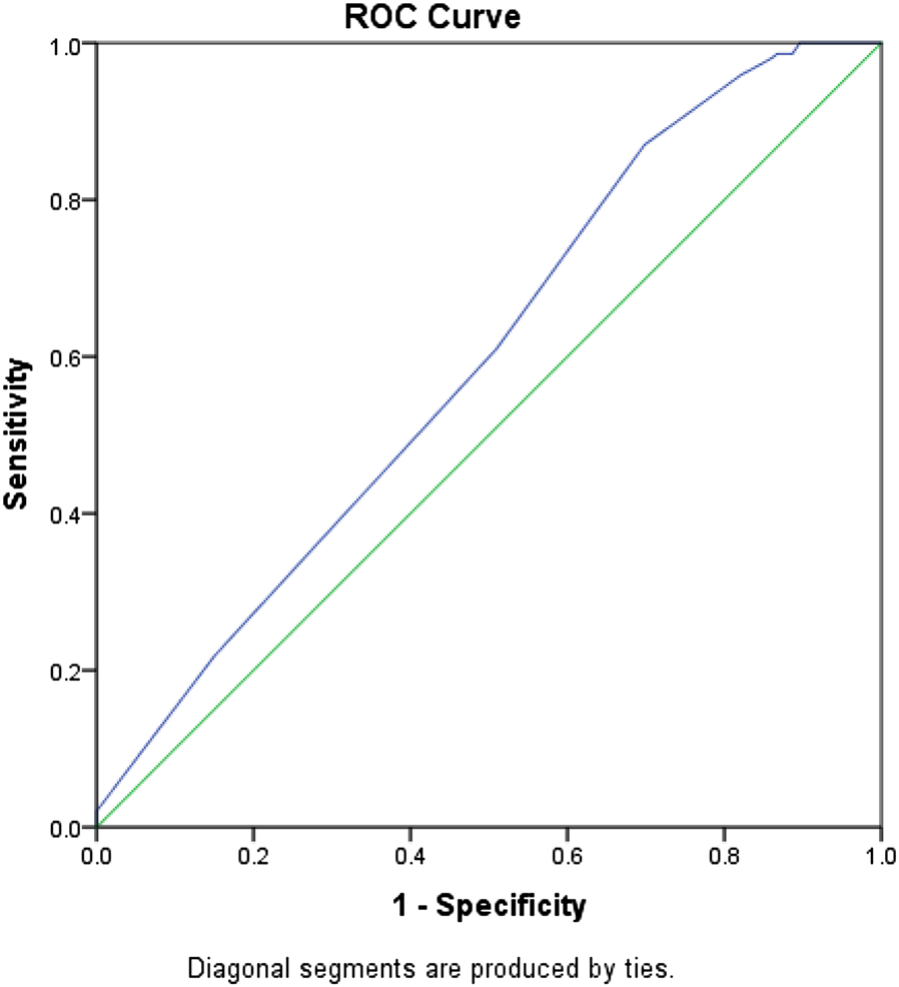
ROC curve to analyze the predictive power of the logistic regression presented in Table 5. The fig indicates AUROC = 0.678 (0.639–0.718) for clinical factors associated rtPA inclusion or exclusion in the non-telestroke population

As shown in Table 2, non-telestroke patients who received rtPA were more likely to be younger (66.8 ± 13.5 vs. 70.8 ± 12), have a lower blood glucose level (171 ± 88 vs. 210.9 ± 126.8), have a lower creatinine 1.3 ± 0.7 vs. 1.6 ± 1.3), a lower INR (1.1 ± 0.1 vs. 1.2 ± 0.4), and present a better ambulatory status at baseline than patients who did not receive rtPA. In the telestroke, patients who received rtPA were more likely to have a worse ambulatory status at presentation and more likely to be directly admitted (77.2% vs. 33.3%). Multivariate analysis reveals four factors associated with rtPA (Table 3). Higher INR [OR, 0.139 (0.029–0.67); 95% CI, P = 0.014] and congestive heart failure [OR, 0.329 (0.124–0.878); 95% CI, P = 0.026] were associated with rtPA exclusion while direct admission [OR = 3.141 (1–9.867); 95% CI, P = 0.050] and being a telestroke patient [OR, 4.87 (1.834–12.928); 95% CI, P = 0.0001] were more associated with rtPA inclusion. The ROC curve for the predictive power of the regression model is presented in Fig. 1. The discriminating capability of the model was very good as shown by the ROC curve, with area under the curve (AUROC) of AUROC = 0.774 (95% CI, 0.712–0.836, P < 0.00). In the non-telestroke (Table 4), older age (> 80 years) [OR, 0.955 (0.922–0.989;95% CI, P = 0.009], higher blood glucose level [OR, 0.994 (0.99–0.999);95% CI, P = 0.0014], higher INR [OR, 0.113 (0.014–0.944);95% CI, P = 0.004], and renal insufficiency [OR, 0.163 (0.033–0.024);95% CI, P = 0.004], were all associated with rtPA exclusion while higher NIH stroke scale [OR, 1.068 (1.009–1.13);95% CI, P = 0.023] was associated with rtPA inclusion. As presented in Fig. 2, the predictive power of the logistic regression was strong. The area under the curve (AUROC) is 0.678 (95% CI, 0.639–0.718, P < 0.01). In the telestroke (Table 5), only higher INR [OR, 0.063 (0.003–1.347) 95% CI, P = 0.077]) was associated with rtPA exclusion and the association was not significant. The predictive model power of the logistic regression was strong (Fig. 3), AUROC = 0.678 (95% CI, 0.639–0.718, P<0.05).

## Discussion

In a diabetic acute ischemic stroke population, patients that present with obesity, directly admitted to emergency department, and received thrombolysis therapy have higher odds of being associated with the telestroke setting. Following the adjustment for comorbidities, the telestroke setting represents the strongest predictor for the administration of thrombolysis therapy. In both telestroke and non-telestroke diabetic acute ischemic stroke patients, direct admission represents a predictor for administration of thrombolysis therapy. In the univariate analysis, non-telestroke diabetic stroke patients who received thrombolysis were more likely to be younger, have a lower blood glucose level, lower creatinine, lower INR, and present with a better ambulatory status at baseline than the patients who did not receive thrombolysis. In the telestroke setting, diabetic acute ischemic stroke patients who received thrombolysis were more likely to have a worse ambulatory status at presentation and more likely to be directly admitted to the emergency department.

In the adjusted analysis for the total diabetic stroke population, only direct admission and being treated in the telestroke setting were independent variables associated with administration of thrombolysis therapy. The non-telestroke setting admitted more diabetic stroke patients, but more were excluded from thrombolysis therapy when compared with the telestroke setting. This may be connected with a higher rate of hospital admission of patients with highly variable clinical risk factors, resulting in the exclusion of more admitted patients from thrombolysis therapy when compared with the telestroke setting. In the adjusted analysis for the non-telestroke setting, age (> 80), higher blood glucose level, and renal insufficiency were all associated with exclusion from thrombolysis. The benefits of thrombolysis therapy have been shown in many studies [20, 22–30]. Findings indicate higher functional dependency in stroke patients older than 80 years that received thrombolysis therapy [31–36]. The observed poor functional outcome appeared to be linked to poorer baseline clinical conditions such as congestive heart failure, ischemic heart disease, and hypertension in older stroke patients. In the current study, our results indicate that pre-stroke functional status, higher blood glucose level, age older than 80, and renal insufficiency were all associated with exclusion from thrombolysis therapy in diabetic stroke patients treated in the non-telestroke setting. These factors have been shown to influence functional outcome in longitudinal studies among elderly stroke patients [20, 37, 38]. Stroke-related mortality is linked to age as a major independent risk factor mainly because elderly acute ischemic patients are more susceptible to complications and have more comorbidities than their younger counterparts [39]. However, advanced age should not be a contraindication for thrombolysis in diabetic stroke patients. Instead, the course of treatment should be decided on a case-by-case basis after a detailed evaluation of existing comorbidities and pre-stroke clinical risks as well as the potential benefits of thrombolytic therapy for each individual old diabetic acute ischemic stroke patient.

A major finding in this study is that our multivariate model predicted a direct association of treatment in the telestroke setting as an independent variable with the highest odds for the inclusion of diabetic stroke patients for thrombolysis therapy. Moreover, following adjustment for baseline demographic and clinical risk factors in the telestroke network, only diabetic stroke patients with higher INR were excluded from thrombolysis, and the effect was not significant. These findings differ from the non-telestroke setting in which diabetic stroke patients with increased age, higher blood glucose level, higher INR, and renal insufficiency were all pre-clinical risk factors that predicted exclusion from thrombolysis therapy. The finding that in the non-telestroke setting, diabetic stroke patients with complicated pre-clinical risk factors were associated with a higher likelihood of exclusion from thrombolysis therapy, suggests a more stringent exclusion criteria when compared with the telestroke setting. Therefore, it is possible that telestroke technology provides a real-world clinical setting that streamlines in-hospital evaluation with less stringent exclusion criteria, allowing stroke neurologist to consult quickly on whether or not administer thrombolysis therapy. This may enable an increase in the rate of use and efficiency of the timeline for administration of thrombolysis in the treatment of diabetic acute ischemic stroke patients.

There are limitations to our study. First, our study is limited by its retrospective design, although data was collected using an established prospective stroke registry, a risk of selection bias is possible. Furthermore, this is unicenter stroke registry and does not allow for the generalization of our findings. Moreover, information about the management of diabetes mellitus (type I or type II) was not included in our analysis. The relatively small groups of patients of diabetic stroke patients did not increase the power of our analysis. The strengths of our study are that in the non-telestroke setting, increased age, higher blood glucose level, renal insufficiency were pre-clinical risk factors that predicted the exclusion from thrombolysis therapy, while only INR predicted a non-significant exclusion from thrombolysis therapy in the telestroke setting. Our multivariate model was able to identify treatment in the telestroke setting as an independent variable with the highest prediction for the inclusion of diabetic stroke patients for thrombolysis therapy. Finally, we found that in older diabetic stroke patients (> 80 years), exclusion maybe linked with pre-treatment functional status that includes history of higher blood glucose level, higher INR, and renal insufficiency.

## Conclusion

Diabetes is not an exclusion criterion for thrombolysis, however, a low rate of thrombolysis therapy has been reported in diabetic acute ischemic stroke patients. More studies are necessary to determine how identified exclusion risk factors in the non-telestroke setting can be improved to provide a real-world clinical setting with less stringent exclusion criteria for thrombolysis therapy.

## Abbreviations

rtPA: recombinant tissue plasminogen activator
TIA: trans ischemic attack
OR: adjusted odd ratio
GWTG: get with the guideline
AHA: American Heart Association
NIH scores: National Institute of Health scores
AUROC: area under the curve
ROC: receiver operator characteristic
CI: confidence interval
CAD: coronary artery disease
MAP: mean arterial pressure
CHF: congestive heart failure
PVD: peripheral vascular disease
GHS: Greenville Health System
